# Nuclear PML expression as a prognostic biomarker in localised clear cell renal cell carcinoma

**DOI:** 10.1002/bco2.70258

**Published:** 2026-07-31

**Authors:** Sakari Kosola, Teemu D. Laajala, Iida Payne, Antti Kukkula, Lassi Luomala, Tuomas Mirtti, Kalle Mattila, Paula Vainio, Maria Sundvall, Panu M. Jaakkola

**Affiliations:** ^1^ Department of Oncology and Radiotherapy, FICAN West Cancer Centre University of Turku and Turku University Hospital Turku Finland; ^2^ Doctoral Programme in Clinical Research (DPCR) University of Turku Turku Finland; ^3^ Department of Mathematics and Statistics University of Turku Turku Finland; ^4^ Research Program in Systems Oncology, Faculty of Medicine University of Helsinki Helsinki Finland; ^5^ Cancer Research Unit, Institute of Biomedicine and FICAN West Cancer Research Laboratories University of Turku Turku Finland; ^6^ iCANDOC Doctoral Programme University of Turku Turku Finland; ^7^ Turku Doctoral Programme of Molecular Medicine (TuDMM) University of Turku Turku Finland; ^8^ Department of Urology University of Helsinki and Helsinki University Hospital Helsinki Finland; ^9^ Department of Pathology University of Helsinki and Helsinki University Hospital Helsinki Finland; ^10^ iCAN‐Digital Precision Cancer Medicine Flagship Helsinki Finland; ^11^ Finnish Cancer Institute Helsinki Finland; ^12^ Comprehensive Cancer Center Helsinki University Hospital Helsinki Finland; ^13^ Department of Pathology Turku University Hospital Turku Finland

**Keywords:** cancer‐specific survival, clear cell renal cell carcinoma, immunohistochemistry, overall survival, prognostic biomarker, promyelocytic leukaemia protein, recurrence

## Abstract

**Objectives:**

To evaluate nuclear promyelocytic leukaemia protein (PML) expression as a prognostic biomarker in localised clear cell renal cell carcinoma (ccRCC), aiming to improve postoperative risk stratification.

**Materials and methods:**

Two independent retrospective cohorts of patients undergoing surgery for localised ccRCC were analysed: an exploratory cohort (*n* = 191; Turku University Hospital) and a validation cohort (*n* = 173; Helsinki University Hospital). Patients underwent surgery between 2005 and 2014 and 2006–2013, respectively. Tissue microarrays from primary tumours were immunohistochemically stained and assessed for nuclear PML expression using both visual scoring and QuPath‐assisted image analysis. Clinical data at the time of surgery and follow‐up data were collected, with a median follow‐up of 8 years in the exploratory cohort and 9 years in the validation cohort. Associations between nuclear PML expression and recurrence‐free survival (RFS), cancer‐specific survival (CSS), overall survival (OS) and diagnostic histopathological characteristics were evaluated.

**Results:**

High nuclear PML expression was associated with improved RFS (*p* = 0.022) and CSS (*p* = 0.027) in the exploratory cohort, with similar findings in the validation cohort in univariable analyses (both *p* < 0.0001). In the validation cohort, high nuclear PML expression remained independently associated with improved RFS (HR 0.38, 95% CI 0.21–0.70; *p* = 0.002), CSS (HR 0.30, 95% CI 0.13–0.68; *p* = 0.004) and OS (HR 0.34, 95% CI 0.18–0.68; *p* = 0.002) after adjustment for established histopathological risk factors in multivariable analyses.

**Conclusions:**

Nuclear PML expression is associated with favourable outcomes in localised ccRCC and may provide additional prognostic information. Independent validation and mechanistic studies are warranted.

## 1 INTRODUCTION

Renal cell carcinoma (RCC) is the most common malignant tumour of the kidney and accounts for approximately 179 000 cancer‐related deaths worldwide annually.^1^ Clear cell renal cell carcinoma (ccRCC) is the predominant histological subtype, representing 70%–75% of cases.^2^ Despite curative‐intent surgery, nearly one‐third of patients with localised disease eventually develop metastatic recurrence.^3^

Several clinical trials have evaluated adjuvant treatment strategies in ccRCC, with most failing to demonstrate consistent improvements in survival outcomes. Updated results from the KEYNOTE‐564 trial showed improved overall survival with adjuvant pembrolizumab; however, three other trials assessing adjuvant and perioperative immune checkpoint inhibitor therapy failed to demonstrate comparable survival benefits.^4^ Although survival in metastatic RCC has improved with targeted therapies and immunotherapy, acquired resistance remains a major clinical challenge, leading to disease progression and death.^5^

Current clinicopathological models and prognostic nomograms provide clinically useful risk stratification, yet their ability to predict individual outcomes remains limited, emphasising the need for robust prognostic and predictive biomarkers.^6^ ccRCC is characterised by substantial intratumoural and intertumoural heterogeneity, as demonstrated by comprehensive genomic and multi‐omic analyses showing metabolic and immune heterogeneity.^7^ No molecular biomarkers are currently incorporated into routine clinical decision‐making in either the adjuvant or metastatic setting.^8^

Loss of function of the von Hippel–Lindau (VHL) protein represents the most common molecular alteration in ccRCC, occurring in over 80% of tumours. VHL inactivation results in constitutive activation of hypoxia‐inducible factors (HIF)‐1α and HIF‐2α, leading to upregulation of downstream target genes, including angiogenic vascular endothelial growth factor (VEGF).^9^ The promyelocytic leukaemia (PML) protein can suppress HIF‐1α translation and neoangiogenesis, whereas PML fusion proteins may cooperate with HIF factors to promote tumour progression.^10^, ^11^ PML was originally identified in acute PML, in which the t(15;17) translocation generates the oncogenic PML–RARα fusion protein.^12^ Subsequently, PML dysregulation has been reported in several solid and haematologic malignancies, supporting a broader role for PML in tumour biology.^13^ PML localises predominantly to nuclear bodies involved in transcriptional regulation, senescence, apoptosis and DNA damage responses.^14^ Although multiple PML isoforms exist, their biological functions remain incompletely characterised.^15^

The role of PML in ccRCC remains controversial. Experimental studies suggest tumour‐suppressive functions through inhibition of mTOR signalling and reduced HIF‐1α translation, thereby limiting angiogenesis.^10^, ^16^, ^17^ In contrast, recent work by Simoni et al. reported that PML is overexpressed in ccRCC and promotes tumour proliferation through inhibition of the tumour suppressor p53.^18^ Furthermore, elevated *PML* mRNA expression has been associated with high‐risk disease and reduced OS in transcriptomic analyses of primary ccRCC cohorts.^19^, ^20^, ^21^ Together, these findings indicate that the functional role of PML in ccRCC remains unresolved.

In this study, we evaluated nuclear PML expression as a prognostic biomarker in localised ccRCC using two independent retrospective patient cohorts.

## 2 MATERIALS AND METHODS

### 2.1 Patient cohorts

The patient cohorts used in this study have been described previously.^22^ Electronic health records were reviewed to identify RCC patients who underwent partial or radical nephrectomy at Turku University Hospital, Finland, between 2005 and 2014. Only patients with localised disease (N0M0) were included. Patients with a prior history of renal cancer, multifocal renal tumours at diagnosis, non‐clear cell histology or missing key clinical data were excluded. An experienced uropathologist (P.V.) reviewed the original pathology reports and selected representative blocks from the surgical specimens for tissue microarray (TMA) construction. Baseline clinical and histopathological data, as well as follow‐up information, were previously collected by K.M. and P.V. The follow‐up cut‐off date for the exploratory cohort was April 2022. The final exploratory cohort included 191 patients.

Baseline clinical variables included age, sex, clinical stage determined by computed tomography (CT) and serum creatinine at the time of surgery. Baseline prognostic features of the primary tumour included tumour size, TNM stage (according to the AJCC 8th edition), histological subtype, tumour grade (four‐tiered ISUP/WHO nuclear grading system), presence of micro‐ and/or macrovascular invasion, histological tumour necrosis, positive surgical margin status, tumour invasion into adjacent anatomical structures and sarcomatoid or rhabdoid differentiation. Follow‐up data included date of recurrence, death or last follow‐up visit, as well as the cause of death (renal cancer‐specific or other causes). Dates and causes of death were curated from Statistics Finland. Postoperative surveillance was conducted according to local clinical practice and included regular thoracic and abdominal CT scans.

A separate validation cohort was obtained from Helsinki University Hospital, Finland. This cohort included patients treated between 2006 and 2013 using identical inclusion and exclusion criteria. The final validation cohort comprised 173 patients with localised ccRCC. Baseline and follow‐up data, as well as histopathological review and TMA construction, were collected as previously described.^22^ The follow‐up cut‐off date was set as September 2019.

### 2.2 TMA

Haematoxylin and eosin (H&E)‐stained surgical slides were digitally reviewed to identify representative tumour and adjacent normal kidney tissue from formalin‐fixed, paraffin‐embedded (FFPE) blocks. These regions were used for TMA construction using the TMA Grand Master system (3DHISTECH, Budapest, Hungary). For each patient, five cores were sampled: two from the tumour centre (classified as cancer centre), two from the tumour margin (classified as cancer border) (Figure S4) and one from adjacent normal kidney tissue. Core diameter was 1.5 mm in the exploratory cohort and 1.0 mm in the validation cohort. In total, 959 and 911 cores were analysed, respectively. TMA slides were scanned at 0.24 μm/pixel resolution using a Pannoramic P1000 slide scanner (3DHISTECH), uploaded to a secure server and visually scored using CaseViewer software (version 2.4, 3DHISTECH). Statistical analyses were performed using cancer centre TMA cores, selected as the primary tumour compartment.

### 2.3 Immunohistochemistry

Immunohistochemical (IHC) staining was performed on FFPE TMA sections using a semi‐automated LabVision Autostainer (Thermo Fisher Scientific). Sections were de‐paraffinised and rehydrated. Heat‐induced epitope retrieval was performed in a microwave oven (7 min at 600 W followed by 7 min at 450 W) in Tris‐EDTA pH 9.0 buffer, followed by a 20‐min cooling period at room temperature (RT). Endogenous peroxidase activity was quenched using hydrogen peroxide. Non‐specific binding was minimised using Normal Antibody Diluent (Immunologic, Arnhem, the Netherlands, BD09‐125). Slides were then incubated with a rabbit monoclonal anti‐PML primary antibody (clone ab179466, Abcam, Cambridge, UK) at a dilution of 1:2000 in antibody diluent for 60 min at RT. Following washes in 0.05 M Tris–HCl pH 7.6 buffer containing 0.05% Tween 20, sections were incubated with a goat anti‐rabbit HRP‐conjugated polymer‐based secondary antibody (BrightVision One‐Step Detection System, Immunologic, DPVR110HRP) for 30 min at RT. Immunoreactivity was visualised using 3,3′‐diaminobenzidine (DAB) (BrightDAB, Immunologic, BS04‐110) for 10 min. Sections were then counterstained with Mayer's haematoxylin for 1 min, dehydrated, cleared and mounted with Pertex mounting medium (Histolab).

Each TMA slide included negative controls (primary antibody omission) and positive control tissues (tonsil, lymph node, testis and liver). Staining was confined to tumour cell nuclei in positive cases, with no cytoplasmic staining observed; therefore, only nuclear staining was included in all analyses. The PML antibody is reported by the manufacturer to recognise multiple PML isoforms (*n* = 12), and therefore, staining represents combined isoform expression.

### 2.4 Cell culture

Human ccRCC 786‐O cells were cultured in RPMI‐1640 supplemented with 10% FBS, 2 mM L‐glutamine, 10 mM HEPES and 1 mM sodium pyruvate. Penicillin–streptomycin (0.5%) was included except during experiments. Cells were maintained at 37°C in 5% CO_2_ and routinely tested negative for mycoplasma contamination.

### 2.5 RNA interference and transfection

Silencer® Select Validated siRNA (ID: s10715; Ambion/Thermo Fisher Scientific, Austin, TX, USA; designated siPML‐1) and Silencer® Select Pre‐designed siRNA (ID: s194692; Ambion/Thermo Fisher Scientific; designated siPML‐2) targeting *PML* were used for transfection. Silencer™ Select Negative Control No. 1 siRNA (Ambion/Thermo Fisher Scientific; designated siNon‐target‐1, 4390843) and Silencer™ Select Negative Control No. 2 siRNA (Ambion/Thermo Fisher Scientific; designated siNon‐target‐2, 4390846) were used as negative controls.

786‐O cells were transfected 24 h after seeding using siRNA at a final concentration of 10 nM with 2.5% DharmaFECT 1 (Dharmacon). Transfection solutions were prepared in Opti‐MEM (Gibco). After 24‐h incubation, the medium was replaced with fresh antibiotic‐free RPMI‐1640 medium. Knockdown efficiency was verified 48 h after transfection by western blotting and immunofluorescence imaging.

### 2.6 Protein extraction and western blotting

Protein extraction and western blotting were performed as previously described by Virtanen et al.^23^ and are detailed in the Supporting Information.

### 2.7 Immunofluorescence staining

Transfected 786‐O cells were fixed with 4% paraformaldehyde for 10 min, washed three times with PBS, permeabilised with 0.25% Triton X‐100 for 10 min and washed three times with PBS at RT. After 1‐h blocking in 3% BSA in PBS buffer at RT, the coverslips were incubated in blocking solution containing anti‐PML antibody (ab179466, Abcam; 1:500) overnight at +4°C. Coverslips were incubated for 1 h in blocking buffer containing Alexa Fluor 488‐conjugated anti‐rabbit secondary antibody (Invitrogen, Thermo Fisher Scientific, Waltham, MA, USA, A‐11034; 1:1000) at RT. Prior to mounting coverslips on slides in Vectashield (Vector Laboratories), the cells were washed three times with PBS at RT, with the last wash containing 1:1000 DAPI (Thermo Fisher Scientific, Waltham, MA, USA, D1306; 1:1000). The cells were imaged using a 3i spinning disk confocal microscope (Yokogawa). Microscopy images were processed in ImageJ software (version 2.14.0) using the sum slices function.

### 2.8 Manual scoring of protein expression

Nuclear PML expression in tumour samples was visually assessed by a single observer (S.K.), blinded to clinical data. Staining intensity was categorised as follows (Figure S1): score 0 (negative/no detectable nuclear staining), score 1 (weak nuclear staining) and score 2 (strong/diffuse nuclear staining). For statistical analysis, scores 0 and 1 were combined into a low‐expression group and compared with score 2 (high expression).

### 2.9 Software‐augmented IHC scoring with QuPath software

QuPath software (version 0.5.1) was used by the same observer (S.K.) for semi‐automated quantification of PML expression in parallel with manual scoring. DAB‐based detection was performed with manual optimisation of stain vector separation to improve discrimination between DAB and haematoxylin signals. Tumour regions were manually annotated by S.K. in each TMA core, excluding stromal, necrotic and adjacent normal tissue. Nuclear detection was performed using the Positive Cell Detection tool^24^ with parameters described in Table S1. A three‐tier scoring system was applied based on the proportion and intensity of positively stained nuclei. Samples with 0%–5% positive nuclei and <1% high‐intensity staining were classified as negative (score 0). Samples with >5% positive nuclei and <30% high‐intensity staining were classified as low expression (score 1). Samples with ≥50% positive nuclei and ≥30% high‐intensity staining were classified as high expression (score 2) (Table S2 and Figure S2). Cut‐off values were defined based on concordance with manual scoring and staining intensity distributions in the exploratory cohort. Scatterplot analysis of QuPath‐derived data was used for visualisation of expression group separation (Figure S3). For statistical analysis, scores were similarly dichotomised into low (0–1) and high (2) expression groups.

### 2.10 Statistical methods

All statistical analyses were performed using R Statistical Software (version 4.5.2).^25^ For each patient, the highest intensity score across TMA cores was used for analysis. Scores 0–1 were combined and compared with score 2 (high expression). Data from visual scoring were used for the primary analysis.

Kaplan–Meier survival curves were plotted to illustrate the association between univariable PML and endpoints using the R packages *survival* (v3.8‐3) and *survminer* (0.5.1). Multivariable Cox proportional hazards models were fitted similarly while adjusting for confounders as presented in Table 2 and Tables S3 and S4. In model fitting for CSS, non‐RCC cause of death was considered as censoring. In model fitting for RFS, any cause of death was considered as censoring.

Complete‐case analysis was used in multivariable models. Missing data were not imputed. The proportional hazards assumptions of the Cox models were verified by manual inspection of Schoenfeld residuals. Kruskal–Wallis rank‐based test and Fisher's exact test were used to assess differences in numeric and ordinal variables, respectively. Fine–Gray competing risk models for RCC‐death versus non‐RCC death specificity were fitted using *cmprsk* (2.2‐12). A two‐sided *p*‐value below 0.05 was considered statistically significant.

### 2.11 Ethical statement

The study was approved by the Institutional Review Boards of Helsinki and Turku University Hospitals (Helsinki University Hospital Ethical Committee, HUS/1040/2018; study permit HUS/419/2018; Turku University Hospital permit T1402_2023; Auria Biobank permission AB24–0317). Informed consent was waived due to the retrospective study design in accordance with the Finnish Act on the Secondary Use of Health Data. All data were de‐identified prior to analysis and handled in compliance with applicable data protection regulations.

## 3 RESULTS

### 3.1 Antibody validation

**FIGURE 1:**
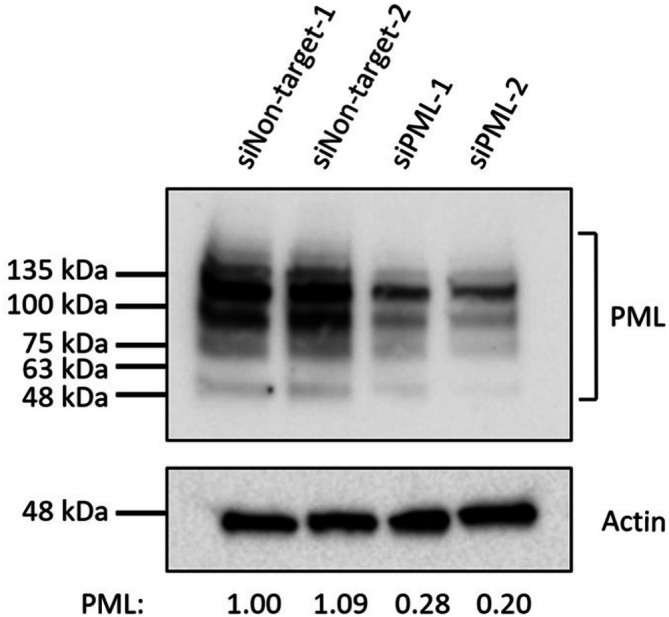
Validation of PML antibody by western blot in 786‐O cells following siRNA knockdown. PML protein levels are shown for knockdown (siPML‐1, siPML‐2) and control (siNon‐target‐1, siNon‐target‐1) samples. Numbers below indicate the expression of PML normalised to the expression level of the loading control actin.

Endogenous PML protein levels were downregulated in 786‐O ccRCC cells after transfection with two independent PML siRNAs (siPML‐1 and siPML‐2), as shown by western blot analysis (Figure 1). All bands were reduced after PML siRNA transfection, demonstrating that the antibody specifically recognised PML, with no evidence of non‐specific signal.

**FIGURE 2:**
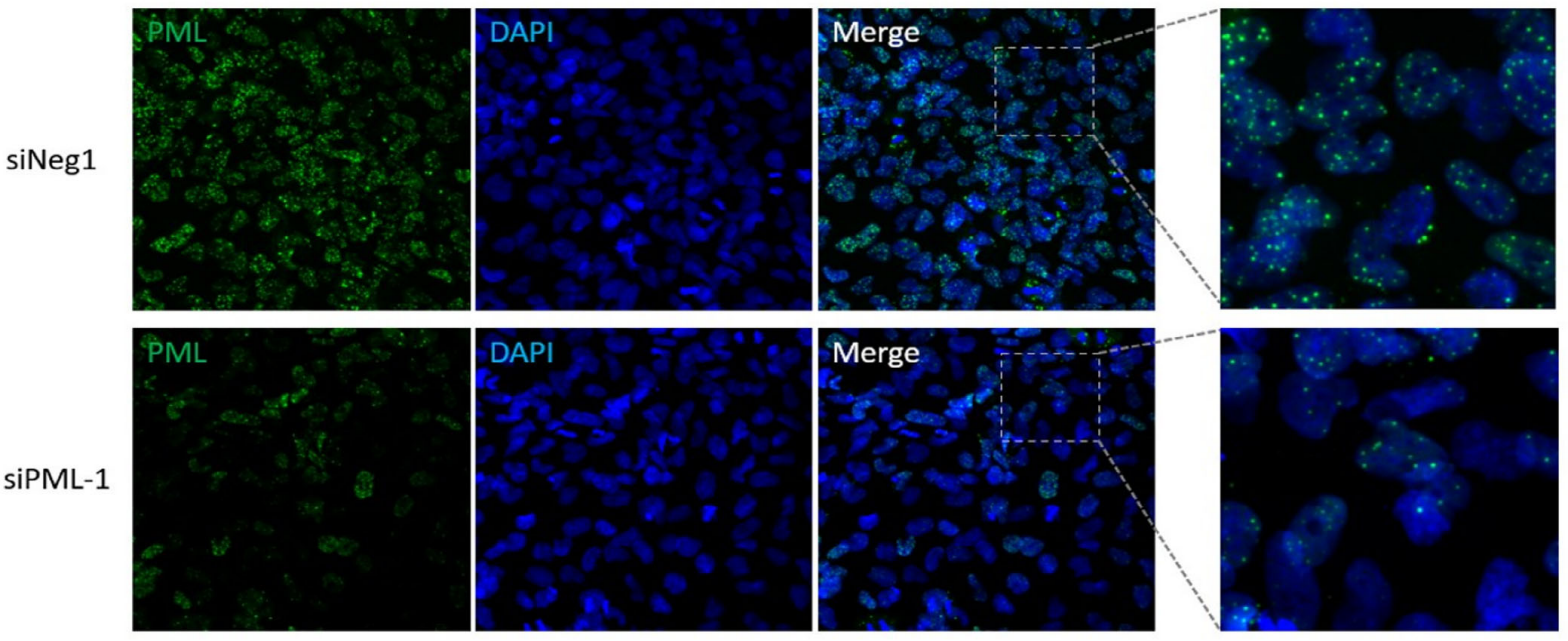
Validation of PML knockdown by immunofluorescence. Nuclear PML is visible in negative control cells (siNon‐target‐1) and significantly reduced in siPML‐1‐transfected 786‐O cells.

Immunofluorescence analysis following PML silencing further confirmed the specificity and efficacy of the antibody, showing loss of the strong PML signal in subnuclear dot‐like structures corresponding to PML nuclear bodies (Figure 2).

### 3.2 IHC‐scoring validation

Visually scored PML IHC expression was compared with QuPath‐guided scoring. Agreement between methods was assessed using Cohen's kappa (Tables S5 and S6). For cancer centre samples, kappa values were 0.72 (0.65–0.79) in the exploratory cohort and 0.92 (0.89–0.96) in the validation cohort, showing a high level of agreement. Similarly, for cancer border samples, agreement was 0.79 (0.72–0.85) and 0.87 (0.79–0.96) in the exploratory and validation cohorts, respectively.

### 3.3 Patient characteristics and treatment outcomes

Baseline patient and tumour characteristics at the time of surgery, along with follow‐up data and endpoint events, are summarised in Table 1.

**TABLE 1:** Patient characteristics in the two cohorts: exploratory (Turku) and validation (Helsinki). Numeric variables are reported as median (IQR), and ordinal variables are reported as absolute units (percentage within column). Kruskal–Wallis rank‐based test was used to test similarity for numeric variables and Fisher's exact test for ordinal values between cohorts.

Variable	Options/units	Exploratory cohort	Validation cohort	*p*
*N*	Patients	191	173	
Age	Years	67 (58–74)	64 (57–72)	0.033
Tumour diameter	mm	60 (40–82)	60 (40–85)	0.863
Sex	Male/female	111 (58%)/80 (42%)	93 (54%)/80 (46%)	0.459
Fuhrman	1	12 (6%)	11 (6%)	0.651
	2	97 (61%)	99 (57%)	
	3	70 (37%)	54 (31%)	
	4	12 (6%)	9 (5%)	
T‐stage	T1	116 (61%)	79 (46%)	<0.001
	T2	50 (26%)	23 (13%)	
	T3 or T4	25 (13%)	71 (41%)	
Sarcomatoid	No/yes	180 (94%)/11 (6%)	162 (94%)/9 (5%)	0.999
	Missing	0 (0%)	2 (1%)	
Necrosis	No/yes	109 (57%)/81 (42%)	121 (70%)/50 (29%)	0.009
	Missing	1 (1%)	2 (1%)	
Microvascular invasion	No/yes	153 (80%)/38 (20%)	137 (79%)/36 (21%)	0.896
	Missing	0 (0%)	0 (0%)	
Macrovascular invasion	No/yes	107 (56%)/20 (10%)	143 (83%)/30 (17%)	0.756
	Missing	64 (34%)	0 (0%)	
Infiltration perirenal fat	No/yes	147 (77%)/19 (10%)	131 (76%)/42 (24%)	0.003
	Missing	25 (13%)	0 (0%)	
Infiltration peripelvic fat	No/yes	129 (68%)/23 (12%)	135 (78%)/38 (22%)	0.12
	Missing	39 (20%)	0 (0%)	
Cause of death	Alive at end of follow‐up	89 (47%)	118 (68%)	<0.001
	RCC death	56 (29%)	45 (26%)	
	Non‐RCC death	46 (24%)	10 (6%)	
Recurrence during follow‐up	No/yes	126 (66%)/65 (34%)	103 (60%)/70 (40%)	0.232
Follow‐up time	Alive, years	9.3 (8.2–11.7)	9.8 (8.7–10.7)	0.750
	RCC death, years	3.9 (2.3–8.3)	4.2 (2.0–5.6)	0.237
	Non‐RCC death, years	5.4 (2.5–8.6)	9.1 (7.3–10.2)	0.019
	Recurrence, years	2.6 (1.5–5.3)	1.5 (0.7–3.2)	0.002
	All patients (until death/censoring), years	8.2 (3.5–10.3)	9.0 (5.7–10.2)	0.055

The median age was higher in the exploratory cohort than in the validation cohort (67 vs. 64 years, *p* = 0.033). T1‐stage tumours were more frequent in the exploratory cohort, whereas T3–T4 tumours were more common in the validation cohort (*p* < 0.001). Tumour necrosis was more prevalent in the exploratory cohort (*p* = 0.009). Perirenal fat invasion was more frequent in the validation cohort (*p* = 0.003), whereas peripelvic fat invasion did not differ between the cohorts (*p* = 0.12).

Missing data differed between the cohorts; in the exploratory cohort, macrovascular invasion was missing in 34%, perirenal fat invasion in 13% and peripelvic fat invasion in 20% of patients. No missing data were present in the validation cohort.

During a median follow‐up exceeding 8 years in both cohorts, recurrence occurred in 65 patients (34%) in the exploratory cohort and 70 patients (40%) in the validation cohort. Non‐RCC‐related deaths occurred in 46 patients in the exploratory cohort compared with 10 in the validation cohort (*p* < 0.001).

### 3.4 Univariable analysis of nuclear PML expression and recurrence‐free, cancer‐specific and overall survival

**FIGURE 3:**
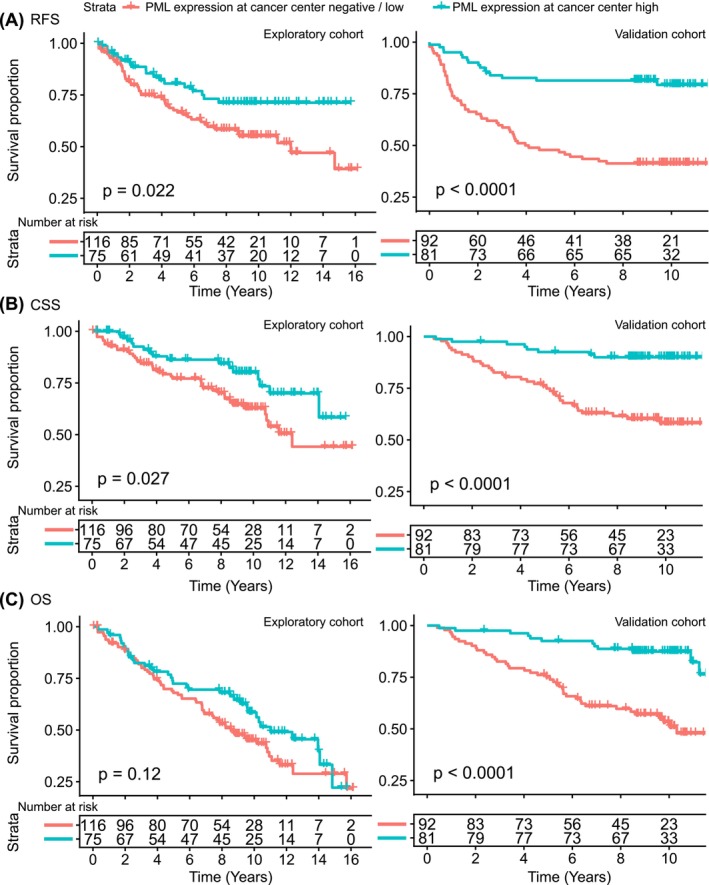
Kaplan–Meier curves with log‐rank *p*‐values for PML high expression versus negative or low expression. Left: Exploratory cohort (Turku). Right: Validation cohort (Helsinki). (A) Recurrence‐free survival. (B) Cancer‐specific survival. (C) Overall survival.

The prognostic significance of PML nuclear expression was assessed using Kaplan–Meier survival analyses for RFS, CSS and OS. Results for both cohorts are shown in Figure 3.

High nuclear PML expression was associated with improved CSS and RFS in both cohorts (Figure 3A,B; *p* < 0.05). For OS, an association with improved survival was observed in the validation cohort (Figure 3C; *p* < 0.0001), whereas this effect was not seen in the exploratory cohort (*p* = 0.12).

### 3.5 Multivariable analyses of nuclear PML expression adjusted for histopathological factors

Multivariable Cox proportional hazards models for RFS are shown in Table 2. Corresponding analyses for CSS and OS are provided in Tables S3 and S4.

**TABLE 2:** Cox proportional hazards model multivariable regression hazard ratio (HR) estimates and 95% confidence interval (CI) with *p*‐values for the recurrence‐free survival (RFS) endpoint. Left: Exploratory cohort. Right: Validation cohort. Identified effects with statistical significance *p* < 0.05 are bolded.

RFS	Multivariable Cox, exploratory cohort			Multivariable Cox, validation cohort		
Variable	*N*	HR estimate [95% CI]	*p*	*N*	HR estimate [95% CI]	*p*
PML negative/low	69	Reference	—	**91**	**Reference**	—
PML high	45	0.788 [0.37, 1.681]	0.538	**80**	**0.383 [0.208, 0.704]**	**0.002**
T‐stage 1	64	Reference	—	78	Reference	—
T‐stage 2	33	1.927 [0.65, 5.711]	0.237	23	1.837 [0.698, 4.837]	0.218
T‐stage 3–4	17	0.099 [0.005, 2.111]	0.139	70	0.954 [0.328, 2.773]	0.931
Fuhrman 1	9	Reference	—	10	Reference	—
Fuhrman 2	55	0.648 [0.136, 3.077]	0.585	98	1.68 [0.224, 12.597]	0.614
Fuhrman 3	43	0.802 [0.167, 3.844]	0.782	54	2.129 [0.268, 16.908]	0.475
Fuhrman 4	7	1.153 [0.186, 7.163]	0.879	9	3.273 [0.363, 29.51]	0.291
No necrosis	**63**	**Reference**	—	121	Reference	—
Necrosis	**51**	**3.902 [1.663, 9.155]**	**0.002**	50	1.64 [0.892, 3.015]	0.112
No microvascular invasion	91	Reference	—	**135**	**Reference**	—
Microvascular invasion	23	1.101 [0.326, 3.721]	0.877	**36**	**3.28 [1.345, 7.995]**	**0.009**
No macrovascular invasion	99	Reference	—	141	Reference	—
Macrovascular invasion	15	9.633 [0.439, 211.602]	0.151	30	0.856 [0.357, 2.054]	0.728
No infiltration peripelvic fat	101	Reference	—	134	Reference	—
Infiltration peripelvic fat	13	2.201 [0.635, 7.627]	0.213	37	1.334 [0.685, 2.596]	0.397
No infiltration perirenal fat	104	Reference	—	129	Reference	—
Infiltration perirenal fat	10	1.143 [0.296, 4.409]	0.846	42	1.664 [0.85, 3.257]	0.137
Tumour maximum diameter (mm)	114	0.998 [0.984, 1.012]	0.792	171	1.006 [0.999, 1.014]	0.111

In the exploratory cohort, tumour necrosis predicted worse RFS (HR 3.90, 95% CI 1.66–9.16, *p* = 0.002), with no effect observed in the validation cohort (HR 1.64, 95% CI 0.89–3.02, *p* = 0.112). Microvascular invasion was not significant in the exploratory cohort (HR 1.10, 95% CI 0.33–3.72, *p* = 0.877) but predicted worse RFS in the validation cohort (HR 3.28, 95% CI 1.35–8.00, *p* = 0.009).

High nuclear PML expression was not significantly associated with RFS in the exploratory cohort (HR 0.79, 95% CI 0.37–1.68, *p* = 0.538) but predicted improved RFS in the validation cohort (HR 0.38, 95% CI 0.21–0.70, *p* = 0.002).

For CSS and OS, tumour necrosis predicted worse outcomes in the exploratory cohort, but not in the validation cohort. Microvascular invasion was not significant in the exploratory cohort but was associated with worse CSS and OS in the validation cohort.

High nuclear PML expression predicted improved CSS and OS in the validation cohort, with no significant association observed in the exploratory cohort.

Competing risk analysis using Fine–Gray models is shown in Table S7. PML expression was not associated with risk of non‐RCC death in either cohort.

## 4 DISCUSSION

In this study, we evaluated nuclear PML protein expression as a prognostic factor in localised ccRCC using two independent retrospective cohorts. To our knowledge, similar analyses have not been previously reported. High nuclear PML expression was consistently associated with favourable oncological outcomes. Specifically, improved RFS and CSS were observed in both cohorts with long‐term follow‐up. In multivariable analyses adjusted for recognised histopathological variables, nuclear PML expression remained independently associated with improved RFS, CSS and OS in the validation cohort, whereas this association was not statistically significant in the exploratory cohort. However, the direction of effect was consistent across cohorts and endpoints. Collectively, these findings suggest that nuclear PML expression may be linked to favourable prognosis in localised ccRCC.

These results are broadly consistent with experimental studies identifying PML as a tumour suppressor in ccRCC using in vitro and in vivo models and human tumour material.^10^, ^16^, ^17^ In contrast, transcriptomic analyses of TCGA‐KIRC and related cohorts have associated high PML mRNA expression with high‐risk disease, advanced stage and poor clinical outcomes, including reduced overall survival.^18^, ^19^, ^20^, ^21^ In addition, Simoni et al. demonstrated that PML protein expression is elevated in ccRCC cell lines and tumours from the CPTAC cohort and may promote tumour cell proliferation while suppressing p53‐dependent senescence.^18^ These divergent findings may reflect biological heterogeneity within ccRCC, differences in tumour stage and methodological differences between transcriptomic and protein‐based analyses. Post‐translational regulation of PML may further contribute to discordance between mRNA and protein expression.^12^ Therefore, transcript abundance does not necessarily reflect functional protein activity. While our findings support a tumour‐suppressive role for PML in localised disease, its role may be context dependent in advanced ccRCC. These observations underscore the need for validation in larger, stage‐stratified cohorts, ideally including multicentre datasets with metastatic and matched primary tumour material. RNA expression data were not available in our study, limiting direct comparison between transcript and protein levels.

The tumour microenvironment and anti‐tumour immune responses play key roles in limiting cancer progression. PML regulates innate immune signalling, type I interferon responses and modulates interferon‐driven transcriptional programmes, thereby contributing to anti‐tumour immunity.^26^, ^27^, ^28^ These functions may underlie its tumour‐suppressive role in localised ccRCC. Wang et al. reported an association between high PML mRNA expression and increased CD4+ T cell, neutrophil and dendritic cell infiltration.^21^ High CD45^+^ leukocyte density has been associated with disease recurrence in localised ccRCC, whereas higher dendritic cell abundance correlates with improved RFS.^29^ However, direct mechanistic evidence linking PML to immune modulation in ccRCC remains limited.

This study has several limitations. First, it is retrospective, and missing data, including histopathological variables, were not imputed. In addition, although replicate tumour cores were analysed for each patient, TMA‐based sampling may underestimate intratumoural heterogeneity in ccRCC. Furthermore, while dichotomisation of IHC expression scores may improve clinical applicability and reflect routine pathological reporting practices, it may also result in some loss of information. Moreover, adjuvant PD‐1‐based therapy was not in clinical use during the study period. Therefore, the predictive value of nuclear PML expression in the context of adjuvant therapy remains unknown and requires prospective evaluation.

Cohort differences should also be considered when interpreting survival analyses. All analyses were performed independently in each cohort without pooling to preserve the external validation design. The stronger association observed in the validation cohort may reflect differences in stage distribution, with a higher proportion of T3–T4 tumours, as well as reduced statistical power in the exploratory cohort due to missing variables and model complexity. Technical factors, including variation in fixation and tissue block age, may also have influenced IHC assessment. In addition, information regarding subsequent cancer therapies after recurrence was not available.

Nuclear PML expression may have prognostic relevance in localised ccRCC, but external validation is needed before clinical implementation. Current clinicopathological risk models provide clinically useful stratification but remain limited, particularly for patients at intermediate risk. Tumour‐intrinsic biomarkers, such as nuclear PML expression, could help refine risk assessment, although their incremental value remains to be defined. Overall, these findings should be considered hypothesis‐generating and warrant validation in contemporary prospective cohorts, particularly in the context of modern adjuvant systemic therapies.

## 5 CONCLUSIONS

Our study suggests that high nuclear PML protein expression is associated with favourable outcomes in localised ccRCC, including improved recurrence‐free and cancer‐specific survival. Further retrospective and prospective studies are needed to validate its prognostic value as a biomarker in ccRCC. In addition, mechanistic studies are warranted to clarify the tumour‐suppressive role of PML in renal cancer biology.

## AUTHOR CONTRIBUTIONS

**Sakari Kosola:** Conceptualization (equal); methodology (equal); investigation (lead); formal analysis (supporting); visualization (equal); writing—original draft (lead); writing—review and editing (equal). **Teemu D. Laajala:** Methodology (equal); software (lead); formal analysis (lead); visualization (equal); data curation (supporting); writing—original draft (supporting); writing—review and editing (equal). **Iida Payne:** Methodology (equal); investigation (supporting); visualization (equal); writing—original draft (supporting); writing—review and editing (supporting). Antti Kukkula: Methodology (equal); investigation (supporting); writing—original draft (supporting); writing—review and editing (supporting). **Lassi Luomala:** Data curation (equal); writing—review and editing (supporting). **Tuomas Mirtti:** Data curation (equal); resources (equal); writing—review and editing (supporting). **Kalle Mattila:** Data curation (equal); writing—review and editing (supporting). **Paula Vainio:** Methodology (equal); resources (equal). **Maria Sundvall:** Conceptualization (equal); methodology (equal); resources (equal); writing—review and editing (equal); supervision (equal); project administration (supporting); funding acquisition (supporting). **Panu M. Jaakkola:** Conceptualization (equal); methodology (equal); resources (equal); writing—review and editing (equal); supervision (equal); project administration (lead); funding acquisition (lead).

## CONFLICT OF INTEREST STATEMENT

Lassi Luomala has received a lecture fee from MSD. Other authors declare no conflicts of interest.

## Supporting information


**Table S1.** Selected settings for the Positive Cell Detection tool in QuPath software (v0.5.1).
**Table S2.** Composite three‐tier scoring system for IHC evaluation based on the proportion and intensity of positively stained nuclei, as detected using the Positive Cell Detection tool in QuPath.
**Table S3.** Cox proportional hazard model multivariable regression hazard ratio (HR) estimates and 95% confidence interval (CI) with p‐values for the Cancer Specific Survival (CSS) endpoint. Left: Exploratory cohort; Right: Validation cohort. Identified effects with statistical significance *p* < 0.05 are bolded.
**Table S4.** Cox proportional hazard model multivariable regression hazard ratio (HR) estimates and 95% confidence interval (CI) with p‐values for the Overall Survival (OS) endpoint. Left: Exploratory cohort; Right: Validation cohort. Identified effects with statistical significance *p* < 0.05 are bolded.
**Table S5.** Agreement between visual scoring (author S.K.) and QuPath‐guided scoring in the exploratory cohort. Cell values are absolute numbers of patients. The reported metric is weighted Cohen's kappa, presented as the estimate (lower–upper confidence interval).
**Table S6:** Agreement between visual scoring (author S.K.) and QuPath‐guided scoring in the validation cohort. Cell values are absolute numbers of patients. The reported metric is weighted Cohen's kappa, presented as the estimate (lower–upper confidence interval).
**Table S7.** Fine–Gray competing risk model for RCC‐specific death and non‐RCC death with subdistribution hazard ratio (HR) estimates. Statistically significant covariates are indicated with bolded text.

**Figure S1.** Representative images of nuclear PML expression in TMA samples. Expression levels were scored as negative (0), low (1), or high (2) according to staining intensity.

**Figure S2.** Representative TMA samples showing negative, low, and high nuclear PML expression. Scale bar: 250 μm. Expression levels were determined using the Positive Cell Detection tool in QuPath.

**Figure S3.** Scatterplot of nuclear PML expression in the exploratory cohort based on QuPath‐guided scoring. The x‐axis represents the percentage of cells with positive nuclear PML, and the y‐axis represents the percentage of cells with high‐intensity nuclear PML. Each point corresponds to a single TMA core (*N* = 932). Samples were classified as low (blue) or high (orange) expression using predefined thresholds of ≥50% positive cells and ≥30% high‐intensity cells. Vertical and horizontal lines indicate these cut‐off values.

**Figure S4.** Representative images of TMA samples showing PML staining in tumour centre (classified as cancer centre) and tumour margin (classified as cancer border) regions. Scale bar: 200 μm.

## Data Availability

The data supporting the findings of this study are available from the corresponding author upon reasonable request. Access to the datasets may be subject to institutional and ethical restrictions from Turku University Hospital and Helsinki University Hospital.
