# Supplementary material for: Nuclear PML expression as a prognostic biomarker in localised clear cell renal cell carcinoma
Source: BJUI Compass. 2026 Jul 31;7(8):e70258. doi: 10.1002/bco2.70258 (PMC13426025; doi:10.1002/bco2.70258)
Supplement: Supplementary file 1 — Table S1. Selected settings for the Positive Cell Detection tool in QuPath software (v0.5.1). Table S2. Composite three‐tier scoring system for IHC evaluation based on the proportion and intensity of positively stained nuclei, as detected using the Positive Cell Detection tool in QuPath. Table S3. Cox proportional hazard model multivariable regression hazard ratio (HR) estimates and 95% confidence interval (CI) with p‐values for the Cancer Specific Survival (CSS) endpoint. Left: Exploratory cohort; Right: Validation cohort. Identified effects with statistical significance p < 0.05 are bolded. Table S4. Cox proportional hazard model multivariable regression hazard ratio (HR) estimates and 95% confidence interval (CI) with p‐values for the Overall Survival (OS) endpoint. Left: Exploratory cohort; Right: Validation cohort. Identified effects with statistical significance p < 0.05 are bolded. Table S5. Agreement between visual scoring (author S.K.) and QuPath‐guided scoring in the exploratory cohort. Cell values are absolute numbers of patients. The reported metric is weighted Cohen's kappa, presented as the estimate (lower–upper confidence interval). Table S6: Agreement between visual scoring (author S.K.) and QuPath‐guided scoring in the validation cohort. Cell values are absolute numbers of patients. The reported metric is weighted Cohen's kappa, presented as the estimate (lower–upper confidence interval). Table S7. Fine–Gray competing risk model for RCC‐specific death and non‐RCC death with subdistribution hazard ratio (HR) estimates. Statistically significant covariates are indicated with bolded text. [file BCO2-7-e70258-s001.docx]

**Supplementary Materials**

**Supplementary Figures**

**Supplementary Figure 1.** Representative images of nuclear PML expression in TMA samples. Expression levels were scored as negative (0), low (1), or high (2) according to staining intensity.

**Supplementary Figure 2.** Representative TMA samples showing negative, low, and high nuclear PML expression. Scale bar: 250 µm. Expression levels were determined using the Positive Cell Detection tool in QuPath.

**Supplementary Figure 3.** Scatterplot of nuclear PML expression in the exploratory cohort based on QuPath-guided scoring. The x-axis represents the percentage of cells with positive nuclear PML, and the y-axis represents the percentage of cells with high-intensity nuclear PML. Each point corresponds to a single TMA core (N = 932). Samples were classified as low (blue) or high (orange) expression using predefined thresholds of ≥50% positive cells and ≥30% high-intensity cells. Vertical and horizontal lines indicate these cut-off values.

**Supplementary Figure 4**. Representative images of TMA samples showing PML staining in tumour centre (classified as cancer centre) and tumour margin (classified as cancer border) regions. Scale bar: 200µm.

**Supplementary Tables**

**Supplementary Table 1.** Selected settings for the Positive Cell Detection tool in QuPath software (v0.5.1).

| **Parameter** | **Value** |
| --- | --- |
| **Detection Image** | **Optical Density Sum** |
| **Requested Pixel Size** | **0.5 µm** |
| **Nucleus Sigma** | **1.5-2 µm** |
| **Minimum Nucleus Area** | **15-20 µm²** |
| **Threshold** | **0.08-0.1** |
| **Max Background Intensity** | **2** |
| **Intensity Threshold +1** | **0.1** |
| **Intensity Threshold +2** | **0.5** |

**Supplementary Table 2.** Composite three-tier scoring system for IHC evaluation based on the proportion and intensity of positively stained nuclei, as detected using the Positive Cell Detection tool in QuPath**.**

| **Score** | **Expression level** | **Criteria** |
| --- | --- | --- |
| **0** | **Negative** | **0-5**% positive nuclei, <**1**% with high-intensity staining |
| **1** | **Low Expression** | >**5**% positive nuclei, <**30**% with high-intensity staining |
| **2** | **High Expression** | ≥**50**% positive nuclei, ≥**30**% with high-intensity staining |

**Supplementary Table 3.** Cox proportional hazard model multivariable regression hazard ratio (HR) estimates and 95% confidence interval (CI) with p-values for the Cancer Specific Survival (CSS) endpoint. Left: Exploratory cohort; Right: Validation cohort. Identified effects with statistical significance p<0.05 are bolded.

| CSS | Multivariable Cox, Exploratory cohort | | | Multivariable Cox, Validation cohort | | |
| --- | --- | --- | --- | --- | --- | --- |
| Variable | N | HR estimate [95% CI] | p | N | HR estimate [95% CI] | p |
| PML negative/low | 69 | Reference | - | **91** | **Reference** | **-** |
| PML high | 45 | 0.709 [0.328, 1.533] | 0.382 | **80** | **0.298 [0.131, 0.678]** | **0.004** |
| T-stage 1 | 64 | Reference | - | 78 | Reference | - |
| T-stage 2 | 33 | 1.634 [0.537, 4.972] | 0.387 | 23 | 1.777 [0.491, 6.433] | 0.381 |
| T-stage 3-4 | 17 | 0.307 [0.031, 3.006] | 0.311 | 70 | 0.947 [0.245, 3.66] | 0.937 |
| Fuhrman 1 | 9 | Reference | - | 10 | Reference | - |
| Fuhrman 2 | 55 | 0.489 [0.105, 2.28] | 0.362 | 98 | 0.89 [0.11, 7.208] | 0.913 |
| Fuhrman 3 | 43 | 0.629 [0.131, 3.018] | 0.563 | 54 | 0.881 [0.103, 7.545] | 0.908 |
| Fuhrman 4 | 7 | 1.273 [0.21, 7.724] | 0.793 | 9 | 1.4 [0.137, 14.317] | 0.777 |
| No necrosis | **63** | **Reference** | **-** | 121 | Reference | - |
| Necrosis | **51** | **4.26 [1.678, 10.816]** | **0.002** | 50 | 1.865 [0.897, 3.876] | 0.095 |
| No microvascular invasion | 91 | Reference | - | **135** | **Reference** | **-** |
| Microvascular invasion | 23 | 0.938 [0.216, 4.08] | 0.932 | **36** | **4.171 [1.48, 11.757]** | **0.007** |
| No macrovascular invasion | 99 | Reference | - | 141 | Reference | - |
| Macrovascular invasion | 15 | 2.89 [0.28, 29.813] | 0.373 | 30 | 0.443 [0.163, 1.208] | 0.112 |
| No infiltration peripelvic fat | 101 | Reference | - | 134 | Reference | - |
| Infiltration peripelvic fat | 13 | 2.46 [0.711, 8.509] | 0.155 | 37 | 2.101 [0.901, 4.899] | 0.086 |
| No infiltration perirenal fat | 104 | Reference | - | 129 | Reference | - |
| Infiltration perirenal fat | 10 | 0.967 [0.214, 4.361] | 0.965 | 42 | 1.883 [0.857, 4.137] | 0.115 |
| Tumour maximum diameter (mm) | 114 | 1.005 [0.991, 1.02] | 0.470 | 171 | 1.009 [0.999, 1.019] | 0.087 |

**Supplementary Table 4.** Cox proportional hazard model multivariable regression hazard ratio (HR) estimates and 95% confidence interval (CI) with p-values for the Overall Survival (OS) endpoint. Left: Exploratory cohort; Right: Validation cohort. Identified effects with statistical significance p<0.05 are bolded.

| OS | Multivariable Cox, Exploratory cohort | | | Multivariable Cox, Validation cohort | | |
| --- | --- | --- | --- | --- | --- | --- |
| Variable | N | HR estimate [95% CI] | p | N | HR estimate [95% CI] | p |
| PML negative/low | 69 | Reference | - | **91** | **Reference** | **-** |
| PML high | 45 | 0.73 [0.394, 1.355] | 0.319 | **80** | **0.335 [0.166, 0.677]** | **0.002** |
| T-stage 1 | 64 | Reference | - | 78 | Reference | - |
| T-stage 2 | 33 | 1.049 [0.416, 2.641] | 0.919 | 23 | 1.605 [0.525, 4.905] | 0.406 |
| T-stage 3-4 | 17 | 0.661 [0.089, 4.934] | 0.686 | 70 | 0.744 [0.229, 2.419] | 0.623 |
| Fuhrman 1 | 9 | Reference | - | 10 | Reference | - |
| Fuhrman 2 | 55 | 0.711 [0.204, 2.483] | 0.593 | 98 | 0.715 [0.156, 3.268] | 0.665 |
| Fuhrman 3 | 43 | 0.818 [0.227, 2.95] | 0.759 | 54 | 0.799 [0.162, 3.927] | 0.782 |
| Fuhrman 4 | 7 | 0.878 [0.168, 4.586] | 0.877 | 9 | 1.039 [0.166, 6.5] | 0.967 |
| No necrosis | **63** | **Reference** | **-** | 121 | Reference | - |
| Necrosis | **51** | **2.372 [1.223, 4.601]** | **0.011** | 50 | 1.61 [0.814, 3.184] | 0.171 |
| No microvascular invasion | 91 | Reference | - | **135** | **Reference** | **-** |
| Microvascular invasion | 23 | 0.913 [0.274, 3.047] | 0.883 | **36** | **5.041 [2.051, 12.387]** | **<0.001** |
| No macrovascular invasion | 99 | Reference | - | 141 | Reference | - |
| Macrovascular invasion | 15 | 1.287 [0.15, 11.069] | 0.818 | 30 | 0.453 [0.185, 1.109] | 0.083 |
| No infiltration peripelvic fat | 101 | Reference | - | 134 | Reference | - |
| Infiltration peripelvic fat | 13 | 1.715 [0.671, 4.383] | 0.260 | 37 | 2.06 [0.933, 4.547] | 0.074 |
| No infiltration perirenal fat | 104 | Reference | - | **129** | **Reference** | **-** |
| Infiltration perirenal fat | 10 | 1.156 [0.352, 3.793] | 0.811 | **42** | **2.163 [1.035, 4.523]** | **0.040** |
| Tumour maximum diameter (mm) | 114 | 1.003 [0.99, 1.015] | 0.699 | 171 | 1.006 [0.996, 1.015] | 0.232 |

**Supplementary Table 5.** Agreement between visual scoring (author S.K.) and QuPath-guided scoring in the exploratory cohort. Cell values are absolute numbers of patients. The reported metric is weighted Cohen’s kappa, presented as the estimate (lower–upper confidence interval)**.**

| Author S.K. | QuPath | | | | Kappa |
| --- | --- | --- | --- | --- | --- |
| **Cancer centre** | **0** | **1** | **2** | **N/A** |  |
| **0** | 3 | 30 | 0 | 0 |  |
| **1** | 0 | 67 | 10 | 0 |  |
| **2** | 0 | 3 | 72 | 0 |  |
| **N/A** | 0 | 1 | 0 | 2 | 0.72 (0.65-0.79) |
| **Cancer border** | **0** | **1** | **2** | **N/A** |  |
| **0** | 0 | 7 | 0 | 0 |  |
| **1** | 0 | 53 | 13 | 0 |  |
| **2** | 0 | 4 | 101 | 0 |  |
| **N/A** | 0 | 0 | 4 | 6 | 0.79 (0.72-0.85) |

**Supplementary Table 6:** Agreement between visual scoring (author S.K.) and QuPath-guided scoring in the validation cohort. Cell values are absolute numbers of patients. The reported metric is weighted Cohen’s kappa, presented as the estimate (lower–upper confidence interval)**.**

| Author S.K. | QuPath | | | | Kappa |
| --- | --- | --- | --- | --- | --- |
| **Cancer centre** | **0** | **1** | **2** | **N/A** |  |
| **0** | 32 | 3 | 0 | 0 |  |
| **1** | 5 | 49 | 3 | 0 |  |
| **2** | 0 | 5 | 76 | 0 |  |
| **N/A** | 0 | 0 | 0 | 0 | 0.92 (0.89-0.96) |
| **Cancer border** | **0** | **1** | **2** | **N/A** |  |
| **0** | 5 | 1 | 0 | 0 |  |
| **1** | 1 | 58 | 5 | 1 |  |
| **2** | 0 | 3 | 99 | 0 |  |
| **N/A** | 0 | 0 | 0 | 0 | 0.87 (0.79-0.96) |

**Supplementary Table 7**. Fine–Grey competing risk model for RCC-specific death and non-RCC death with subdistribution hazard ratio (HR) estimates. Statistically significant covariates are indicated with bolded text.

| Fine–Grey models | Exploratory cohort | | Validation cohort | |
| --- | --- | --- | --- | --- |
| Variable | HR estimate [95% CI] | p | HR estimate [95% CI] | p |
| RCC-specific death | | | | |
| PML negative/low | Reference | | Reference | |
| PML high | 0.7987 [0.4445, 1.4352] | 0.452 | **0.3226 [0.1393, 0.7471]** | **0.008** |
| Fuhrman 1 | Reference | | Reference | |
| Fuhrman 2 | 0.8666 [0.2066, 3.6359] | 0.845 | 0.8739 [0.1043, 7.3257] | 0.901 |
| Fuhrman 3 or 4 | 1.6161 [0.3843, 6.7960] | 0.512 | 1.5140 [0.1674, 13.6897] | 0.712 |
| T-stage 1 | Reference | | Reference | |
| T-stage 2 | 1.8216 [0.7587, 4.3735] | 0.180 | 1.7157 [0.4093, 7.1920] | 0.460 |
| T-stage 3 or 4 | 1.8614 [0.7740, 4.4768] | 0.165 | **3.1184 [1.0964, 8.8695]** | **0.033** |
| Male (vs. Female) | 0.7796 [0.4400, 1.3813] | 0.394 | 1.0101 [0.5208, 1.9593] | 0.976 |
| Tumour diameter (mm) | **1.0128 [1.0034, 1.0223]** | **0.007** | 1.0080 [0.9976, 1.0184] | 0.132 |
| non-RCC death | | | | |
| PML negative/low | Reference | | Reference | |
| PML high | 0.7756 [0.4006, 1.5016] | 0.451 | 0.5673 [0.1259, 2.5574] | 0.461 |
| Fuhrman 1 | Reference | | Reference | |
| Fuhrman 2 | 0.7734 [0.2166, 2.7613] | 0.692 | 0.6266 [0.0994, 3.9496] | 0.619 |
| Fuhrman 3 or 4 | 0.5739 [0.1508, 2.1838] | 0.415 | 1.3623 [0.1791, 10.3599] | 0.765 |
| T-stage 1 | Reference | | Reference | |
| T-stage 2 | 0.2650 [0.0632, 1.111] | 0.069 | 3.2334 [0.1891, 55.2758] | 0.418 |
| T-stage 3 or 4 | 0.6666 [0.1843, 2.4116] | 0.537 | 2.7279 [0.4129, 18.0245] | 0.298 |
| Male (vs. Female) | 1.5635 [0.7719, 3.1671] | 0.215 | 0.5947 [0.01489, 2.3750] | 0.462 |
| Tumour diameter (mm) | 0.9928 [0.9766, 1.0093] | 0.389 | **0.9709 [0.9468, 0.9957]** | **0.022** |

**Supplementary Data**

**Supplementary Methods**

**Protein extraction and western blotting**

Cells were lysed with lysis buffer containing 1% Triton X-100, 10 mM Tris-HCl (pH 7.4), 1 mM EDTA, 150 mM NaCl, and 10 mM NaF supplemented with 2% complete EDTA-free protease inhibitor cocktail (Roche), 10 mM Na_4_P_2_O_7_, and 1 mM Na_3_VO_4_.

Equal amounts of protein (20 µg) from the supernatants were separated on 4–15% SDS-PAGE gels (Bio-Rad). The separated proteins were transferred onto a nitrocellulose membrane (Sartorius). Nonspecific binding to the membrane was blocked by 1 h incubation in TBST (10 mM Tris-HCl, pH 7.4, 150 mM NaCl, 0.05% Tween-20) buffer containing 5% non-fat dry milk and 1% BSA (Sigma-Aldrich). Membranes were incubated overnight at +4°C with antibodies for PML (ab179466, Abcam; 1:1000) and actin (sc-47778, Santa Cruz Biotechnology, Dallas, TX, USA; 1:1000) diluted in TBST containing 5% BSA (Sigma-Aldrich). Membranes were incubated for 1 h at RT with peroxidase-conjugated goat anti-rabbit IgG (A16104, Invitrogen, Waltham, MA, USA; 1:9000) or peroxidase-conjugated goat anti-mouse IgG (A16072, Invitrogen; 1:9000) diluted in TBST containing 5% milk and 1% BSA (Sigma-Aldrich).

Bound secondary antibody was visualised by Clarity™ Western ECL Substrate kit (#1705060, Bio-Rad) and captured with ChemiDoc™ Touch Gel Imaging System (Bio-Rad). Densitometry was performed using the Image Lab software (v6.1, Bio-Rad).
