# Supplementary figures and images for: Nuclear PML expression as a prognostic biomarker in localised clear cell renal cell carcinoma
Source: BJUI Compass. 2026 Jul 31;7(8):e70258. doi: 10.1002/bco2.70258 (PMC13426025; doi:10.1002/bco2.70258)

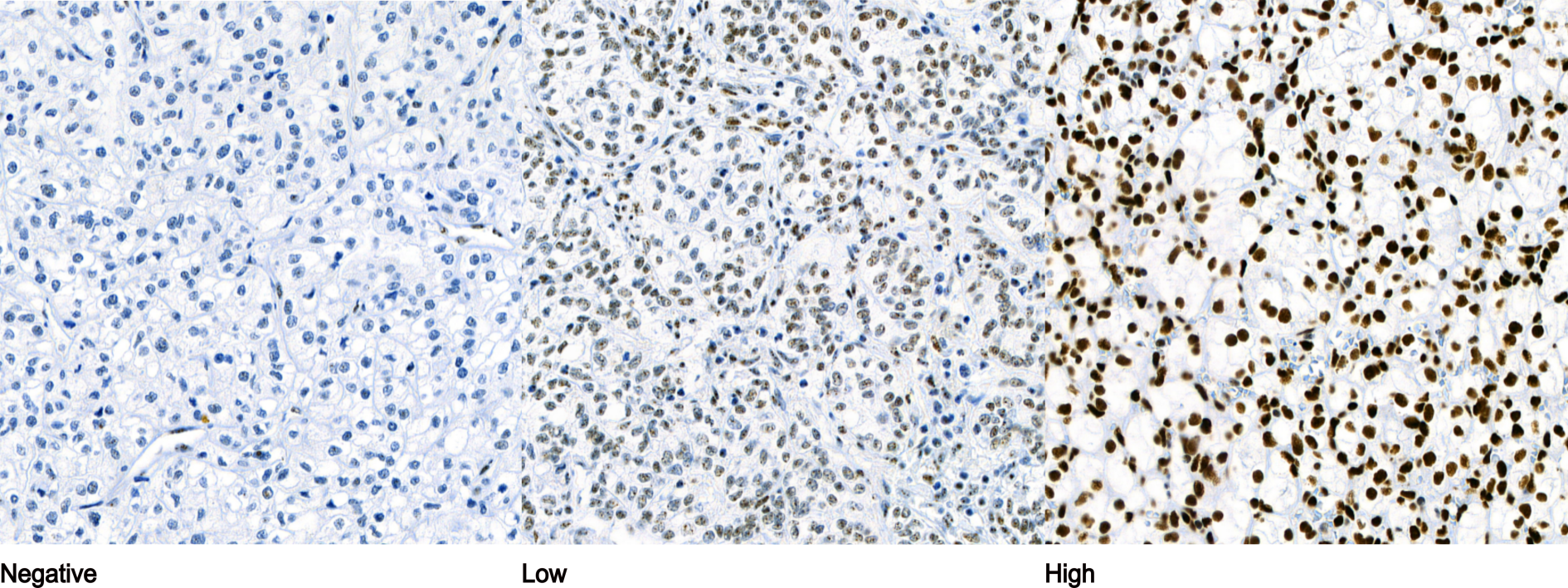

Supplement: Supplementary file 2 — Figure S1. Representative images of nuclear PML expression in TMA samples. Expression levels were scored as negative (0), low (1), or high (2) according to staining intensity. [file BCO2-7-e70258-s002.tiff]

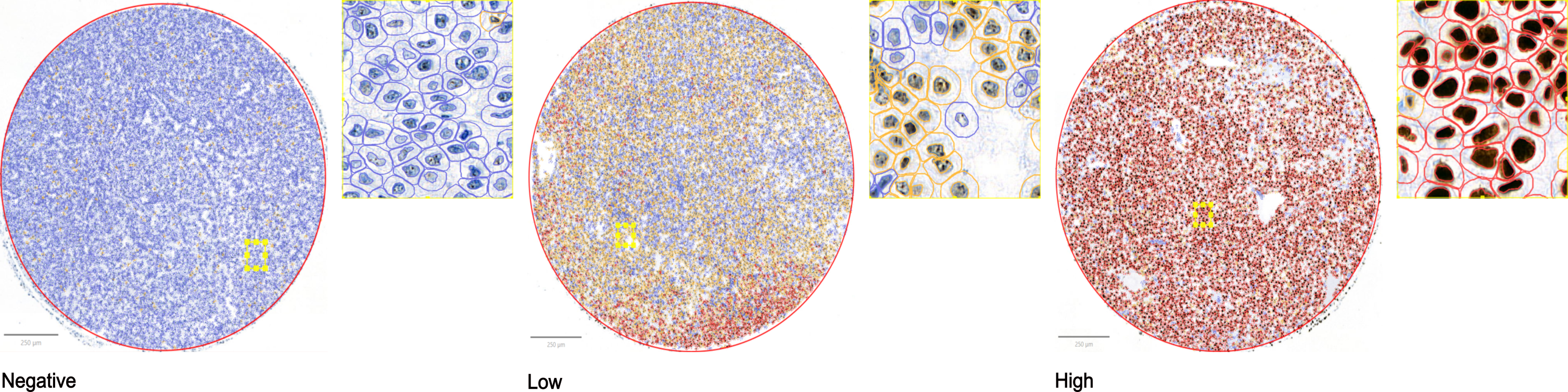

Supplement: Supplementary file 3 — Figure S2. Representative TMA samples showing negative, low, and high nuclear PML expression. Scale bar: 250 μm. Expression levels were determined using the Positive Cell Detection tool in QuPath. [file BCO2-7-e70258-s004.tiff]

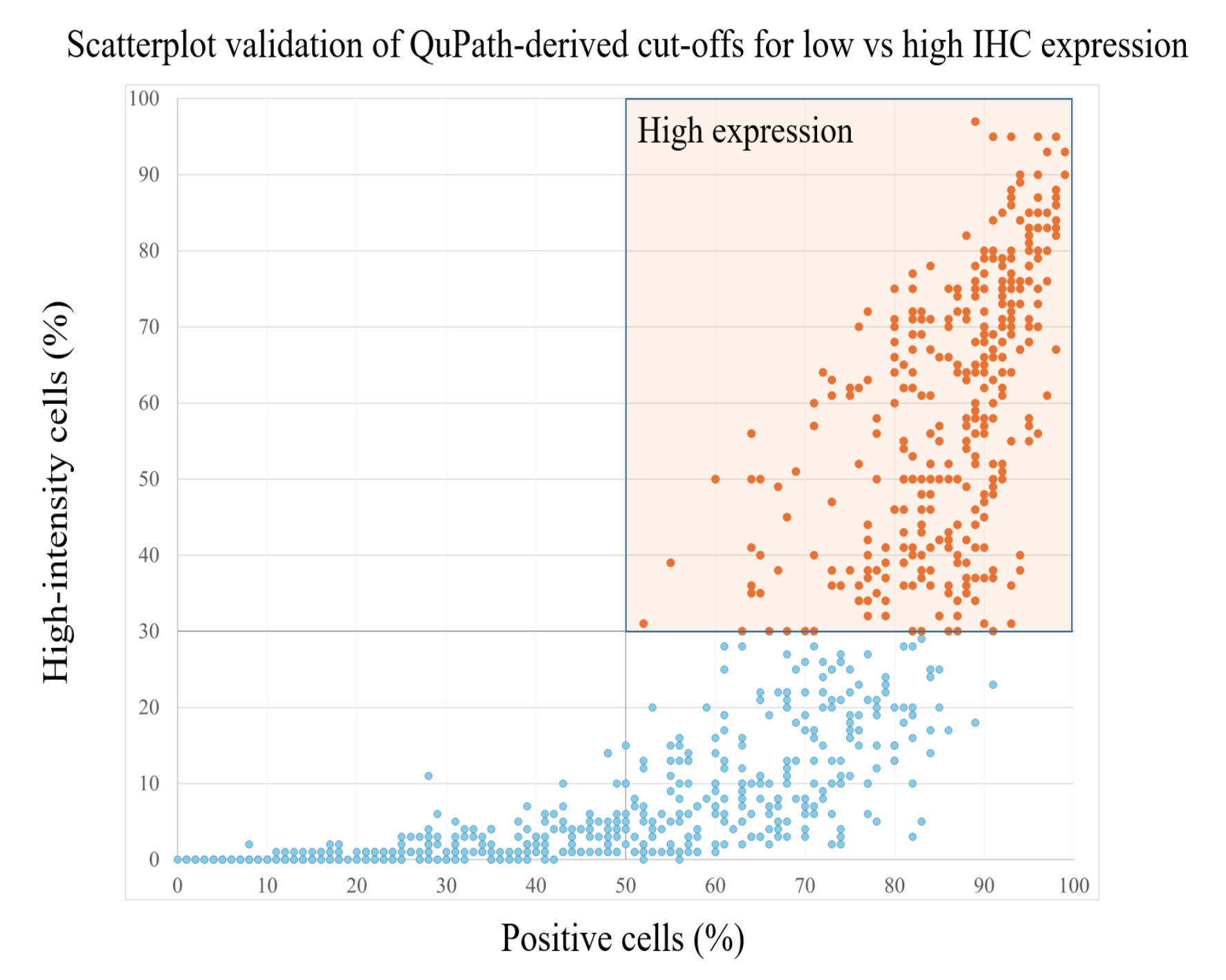

Supplement: Supplementary file 4 — Figure S3. Scatterplot of nuclear PML expression in the exploratory cohort based on QuPath‐guided scoring. The x‐axis represents the percentage of cells with positive nuclear PML, and the y‐axis represents the percentage of cells with high‐intensity nuclear PML. Each point corresponds to a single TMA core (N = 932). Samples were classified as low (blue) or high (orange) expression using predefined thresholds of ≥50% positive cells and ≥30% high‐intensity cells. Vertical and horizontal lines indicate these cut‐off values. [file BCO2-7-e70258-s005.tiff]

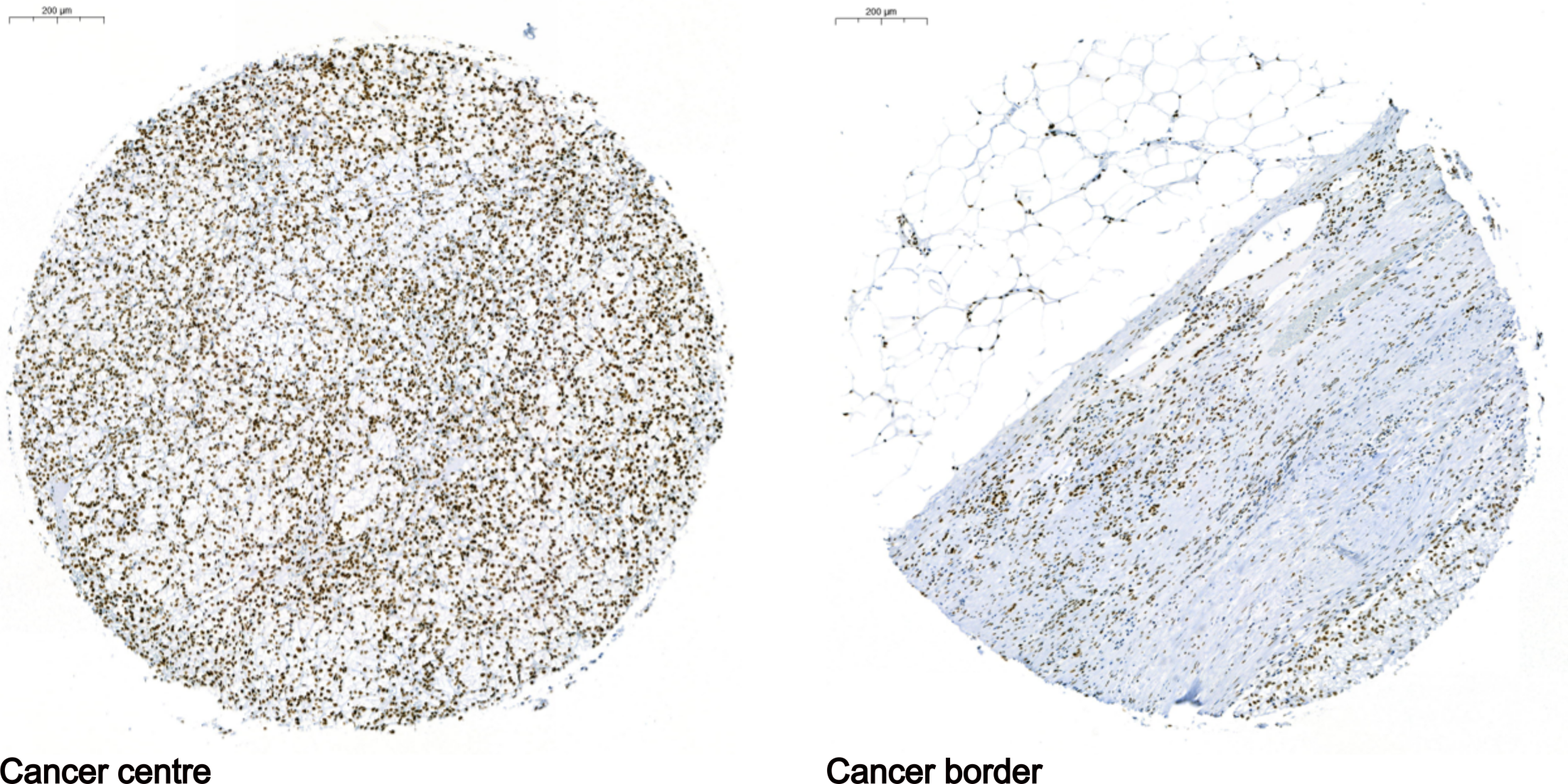

Supplement: Supplementary file 5 — Figure S4. Representative images of TMA samples showing PML staining in tumour centre (classified as cancer centre) and tumour margin (classified as cancer border) regions. Scale bar: 200 μm. [file BCO2-7-e70258-s003.tiff]
